# Detecting Rapid Spread of SARS-CoV-2 Variants, France, January 26–February 16, 2021

**DOI:** 10.3201/eid2705.210397

**Published:** 2021-05

**Authors:** Stéphanie Haim-Boukobza, Bénédicte Roquebert, Sabine Trombert-Paolantoni, Emmanuel Lecorche, Laura Verdurme, Vincent Foulongne, Christian Selinger, Yannis Michalakis, Mircea T. Sofonea, Samuel Alizon

**Affiliations:** Cerba Laboratory, Saint Ouen L’Aumône, France (S. Haim-Boukobza, B. Roquebert, S. Trombert-Paolantoni, E. Lecorche, L. Verdurme);; CHU de Montpellier, Montpellier, France (V. Foulongne);; IRD, Montpellier (C. Selinger); CNRS, Montpellier (Y. Michalakis, S. Alizon);; Université de Montpellier, Montpellier (M.T. Sofonea)

**Keywords:** virus, epidemiology, variants, RT-PCR, screening, statistical modelling, GLM, transmission, COVID-19, coronavirus disease, SARS-CoV-2, severe acute respiratory syndrome coronavirus 2, viruses, respiratory infections, zoonoses, France

## Abstract

Variants of severe acute respiratory syndrome coronavirus 2 raise concerns regarding the control of coronavirus disease epidemics. We analyzed 40,000 specific reverse transcription PCR tests performed on positive samples during January 26–February 16, 2021, in France. We found high transmission advantage of variants and more advanced spread than anticipated.

Since the end of 2020, at least 3 strains, or “variants,” of severe acute respiratory syndrome coronavirus 2 (SARS-CoV-2) bearing a high number of mutations have been associated with rapid epidemic spread in the United Kingdom (lineage B.1.1.7) (*1*), South Africa (lineage B.1.351) (*2*), and Brazil (lineage P.1) (*3*). Because of their increased transmissibility (*4*; E. Volz et al., unpub. data, https://www.medrxiv.org/content/10.1101/2020.12.30.20249034v2; A.S. Walker et al., unpub. data, https://www.medrxiv.org/content/10.1101/2021.01.13.21249721v1) and potential ability to evade host immunity (*5*; S. Cele et al., unpub. data, https://www.medrxiv.org/content/10.1101/2021.01.26.21250224v1), monitoring these variants is crucial in the context of mass vaccination.

In France, beginning February 5, 2021, every sample that tested SARS-CoV-2–positive by reverse transcription PCR (RT-PCR) underwent an additional variant-specific RT-PCR with probes targeting the Δ69–70 deletion and the N501Y mutation, both in the spike glycoprotein. Both targets are present in lineage B.1.1.7. For lineages B.1.351 and P.1, only the N501Y mutation is present. If only the Δ69–70 deletion is detected, the infection might be caused by another variant or by a wild-type strain with a deletion. Finally, if neither target is detected, the infection is considered to be caused by a wild-type strain. These tests are cheaper and easier to implement than full-genome sequencing, which enables their rapid deployment on a wide scale. We report the results of this testing program.

## The Study

RT-PCR testing for SARS-CoV-2 strains was conducted using 2 assays, VirSNiP SARS-CoV-2 Spike del+501 (TIB Molbiol, https://www.tib-molbiol.de) and ID SARS-CoV-2/UK/SA Variant Triplex (ID Solutions, https://www.id-solutions.fr), which target the Δ69–70 deletion and N501Y mutation. We performed tests on 42,229 positive samples collected during January 26–February 16, 2021, from 40,777 persons from 12 regions in France. Most samples came from the general population, and 3,323 (7.9%) samples came from hospitals. For the 1,397 patients for whom multiple tests were performed, only the first test was considered. We only included data from persons 5–80 years of age to minimize the weight of preschool children and persons living in aged-care facilities in our analysis. Finally, we removed persons whose age or testing region was unknown. This study was approved by the Institutional Review Board of the CHU of Montpellier and is registered at ClinicalTrials.gov (identifier NCT04738331).

**Figure 1:**
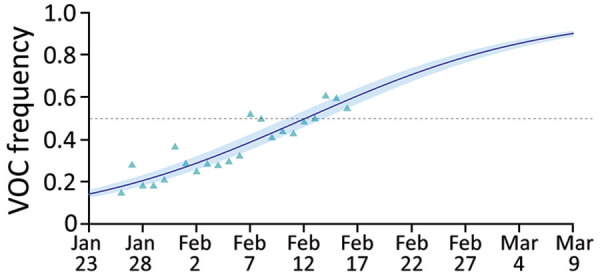
Estimated variants frequency kinetics in study of rapid spread of severe acute respiratory syndrome coronavirus 2 spread, France, January 26–February 16, 2021. Triangles indicate the general linear model–fitted values, line indicates output of the logistic growth model estimation, and shading indicates 95% CIs. Overall estimated transmission advantage of the variants (with respect to the wild-type reproduction number) is 50 (95% CI 38%–64%) (Appendix 2). VOC, variant of concern.

Overall, we analyzed 35,208 SARS-CoV-2–positive samples from the same number of persons (Appendix 1 Table 1). Results of 6,702 (19%) variant-specific RT-PCR tests were uninterpretable, mainly because of an insufficient amplification of the control, which targets the SARS-CoV-2 N gene. These results were treated as missing in the analyses. Given that most of the variants were B.1.1.7 (Appendix 2 Figure 1), we grouped all samples bearing the N501Y mutation into a broader class of variant-positive.

**Figure 2:**
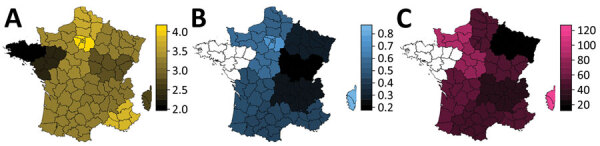
Regions of France in study of rapid spread of SARS-CoV-2 variants, January 26–February 16, 2021. For each region, we show the log_10_ of the number of tests analyzed (A), the estimated total variant frequency by February 16, 2021 (B), and the estimated percentage transmission advantage (of the variant strains relative to the wild-type strain (C). Additional details in Appendix 2 Figure 5.

We used a generalized linear model (GLM) to analyze the binary strain variable (with values wild-type or variant). The covariates were the patient’s age, the RT-PCR kit used for variant detection, the sampling date, and the geographic region from which the sample originated (Appendix 2). By using a type-II analysis of variance, we found that all covariates except the type of RT-PCR kit to be significant (Table 1). In particular, the proportion of variants increased with date and decreased with age (Appendix 2 Figure 2) and hospital origin.

**Table 1:** Risk for variant detection estimated using the general linear model in study of rapid spread of severe acute respiratory syndrome coronavirus 2 variants, France, January 26–February 16, 2021*

Covariate	OR	2.5% CI	97.5% CI
Date, per day	**1.07**	1.03	1.11
Age, per year	**0.993**	0.992	0.995
Kit 2	0.94	0.93	1.16
Nonhospital location	**1.25**	1.13	1.39

*For categorical variables, reference values are the other kit (1) and the hospital setting. Bold indicates statistical significance. OR, odds ratio.

To investigate the temporal trends, we fitted a logistic growth model to the fitted values of an analogous GLM only on the data from general population samples (Appendix 2). Assuming that variations in frequencies are driven by transmission advantages, we found that variants have a 50% (95% CI 37%–64%) transmission advantage over wild-type strains (Figure 1).

The analysis of variance already showed that variant frequency varied across regions (Table 1). We performed the logistic growth fit at the local level for regions for which adequate data was available (Figure 2). The growth advantage of the variant was more pronounced in some regions. In Ile-de-France, more than half of infections already appeared to be caused by the variants by February 16, whereas in other regions, such as Burgundy, this proportion would not be reached until March 2021. However, some regions were less well represented in this analysis, which could affect local estimates.

Finally, we investigated the correlation between the increase in variant frequency among positive tests in a region and the temporal reproduction number, denoted R_t_, in that same region. R_t_ was estimated from coronavirus disease intensive care unit admission data by using the EpiEstim method (*6*) with a serial interval from Nishiura et al. (*7*), as described in Reyné et al. (unpub. data, https://www.medrxiv.org/content/10.1101/2020.12.05.20244376v1) (Appendix 2). We used the Spearman rank correlation test and found a positive but nonsignificant trend (ρ = 0.50; p = 0.09) (Appendix 2 Figure 3).

## Conclusions

We used 2 variant-specific RT-PCR tests to detect the fraction of infections caused by SARS-CoV-2 lineages B.1.1.7, B.351, and P.1 in regions in France during January 25–February 16, 2021. We did not find any significant difference between the 2 specific RT-PCR kits used, suggesting that similar data collected in France could be pooled. Our results have several practical implications.

In general, we found that many infections screened were caused by variants, especially B.1.1.7, and the trend increased over time. On the basis of our estimates, by February 16, 2021, more than half of SARS-CoV-2 infections in France could have been caused by variants, although with pronounced spatial heterogeneity. In a conservative scenario, where all uninterpretable tests were assumed to be caused by the wild type, most infections would have been caused by variants by the end of week 7 of 2021, and the estimated variants transmission advantage was 36% (95% CI 26%–48%) (Appendix 2 Figure 4).

Variant-positive samples originated from significantly younger patients, which is consistent with an earlier report (E. Volz et al., unpub. data) but contrasts with Davies et al. (*4*). Our analysis did not enable us to discriminate between epidemiologic effects (e.g., if variants’ transmission chains were seeded in different populations than the wild types), sampling biases, or biologic effects. Additional data from RT-PCR amplification cycles could provide useful insights. Finally, earlier reports have found variant proportion to be associated with higher basic reproduction number (*4*; E. Volz et al., unpub. data). We found such a trend among regions in France, but it is not statistically significant.

A limitation of this study is that, in spite of its intensity, the sampling was performed retrospectively, which could generate biases if, for instance, transmission chains associated with variants were increasingly sampled. However, we found that samples that originated in hospitals were associated with a lower variant detection. Because testing in the general population is usually performed a week after infection and hospital admissions occur ≈2 weeks after infection (M.T. Sofonea et al., unpub. data, https://www.medrxiv.org/content/10.1101/2020.05.22.20110593v1), we expect hospital data would reflect an older state of the epidemic than screening data. RT-PCR does not have the resolution of full-genome sequencing, and other variants of concern could be underestimated or missed with this approach. However, the time scale considered and the relatively slow evolutionary rate of SARS-CoV-2 make this approach appropriate to monitor variant spread. Furthermore, next-generation sequencing performed on 48 samples showed a strong consistency with the specific RT-PCR tests (Cohen κ of 1 for the TIB Molbiol test and 0.87 or 0.88 for the ID Solutions test depending on the variant; data not shown).

These results illustrate that variant-specific RT-PCRs are an option for SARS-CoV-2 epidemic monitoring because of their affordability and rapid deployment. They also demonstrate that SARS-CoV-2 variants spread in France was faster than anticipated (L.D. Domenico et al., unpub. data, https://www.medrxiv.org/content/10.1101/2021.02.14.21251708v1), which stresses the importance of swift public health responses.

Appendix 1 Additional data used in study of rapid spread of SARS-CoV-2 variants, France, January 26–February 16, 2021.

Appendix 2 Additional information about detection of rapid spread of SARS-CoV-2 variants, France, January 26–February 16, 2021.
