# Supplementary material for: Detecting Rapid Spread of SARS-CoV-2 Variants, France, January 26–February 16, 2021
Source: Emerg Infect Dis. 2021 May;27(5):1496–9. doi: 10.3201/eid2705.210397 (PMC8084510; doi:10.3201/eid2705.210397)
Supplement: Appendix 2 — Additional information about detection of rapid spread of SARS-CoV-2 variants, France, January 26–February 16, 2021. [file 21-0397-Techapp-s2.pdf]

Article DOI: https://doi.org/10.3201/eid2705.210397

Detecting Rapid Spread of SARS-CoV-2 Variants, France, January 26–February 16, 2021

Appendix 2

Supplementary Methods

Generalized Linear Model (GLM)

The main model was performed by using a GLM assuming a binomial distribution, where the variable of interest was the binary variable strain (i.e., wild type or variant) and the explanatory variables were the sampling date (integer), the individual age (integer), the test kit used (boolean), the location of the sampling (boolean), and the region (factor). We further added an interaction between the region and the date. Odds ratios were computed by estimating a likelihood profile. We use a type II error for the analysis of variance given the uneven sampling between regions (the analysis of variance function in the car package of R).

Logistic Growth Fitting

We used the fitted values of the GLM model applied to the data after removing samples that came from hospitals (the sampling location effect was also obviously removed from the model) to perform the inference of a 2-parameter logistic growth kinetic curve: $f(t) = \left(1 + e^{-\sigma(t-\tau)}\right)^{-1}$, where $f(t)$ is the frequency of the variants in the new infections at time t, σ is the relative growth rate of the variants and τ is the time at which f reaches 1⁄2. This method is indeed more appropriate to deal with temporal auto-correlation biases in proportion time series (*1*; E. Volz et al., unpub. data, https://www.medrxiv.org/content/10.1101/2020.12.30.20249034v2).

The parameter estimation was performed by using the drc package in R at the national and regional level (for regions with ≥1,000 samples). The CI of the fitted curves rely on those of the estimated date of reaching half proportion of new infections (τ). The unitless estimated

transmission advantage, ETA, is expressed in terms of multiplicative gain in reproduction number with respect to that of the wild type, such that $R_{variant} = (1 + ETA) * R_{wildtype}$.

Its calculation was made by solving the Euler-Lotka equation ($R_{variant} \int_0^\infty e^{-\sigma t} w(t) dt = 1$) assuming a serial interval *w* following a Weibull distribution with a mean and SD of 4.8 and 2.3 days (*2*) and a constant equal to 1. The CI relies on those of the estimated relative growth rate.

Reproduction Number and Variant Increase

The reproduction number was estimated by using the method described in Reyné et al. (unpub. data, https://www.medrxiv.org/content/10.1101/2020.12.05.20244376v1). In brief, intensive care unit admission data was collected from https://www.data.gouv.fr/fr/datasets/donnees-hospitalieres-relatives-a-lepidemie-de-covid-19 and the reproduction number was then computed for each region in France by using the EpiEstim package in R by setting the serial interval to that reported by Nishiura et al. (*2*).

To estimate the increase in the proportion of variants among positive tests, we performed a GLM with a binomial distribution to explain the type of infection (wild type or variant) as a function of 2 factors (sampling date and individual age). This was done only on data collected outside hospital settings. The regression coefficients were used to perform a Spearman's rank correlation with the most recent time point reproduction number estimate (February 16, 2021) (Appendix 2 Figure 4).

References

1. Davies NG, Abbott S, Barnard RC, Jarvis CI, Kucharski AJ, Munday J, et al. Estimated transmissibility and severity of novel SARS-CoV-2 variant of concern 202012/01 in England. Science. 2021;eabg3055 [Epub ahead of print]. https://doi.org/10.1126/science.abg3055

2. Nishiura H, Linton NM, Akhmetzhanov AR. Serial interval of novel coronavirus (COVID-19) infections. Int J Infect Dis. 2020;93:284–6. https://doi.org/10.1016/j.ijid.2020.02.060 PubMed

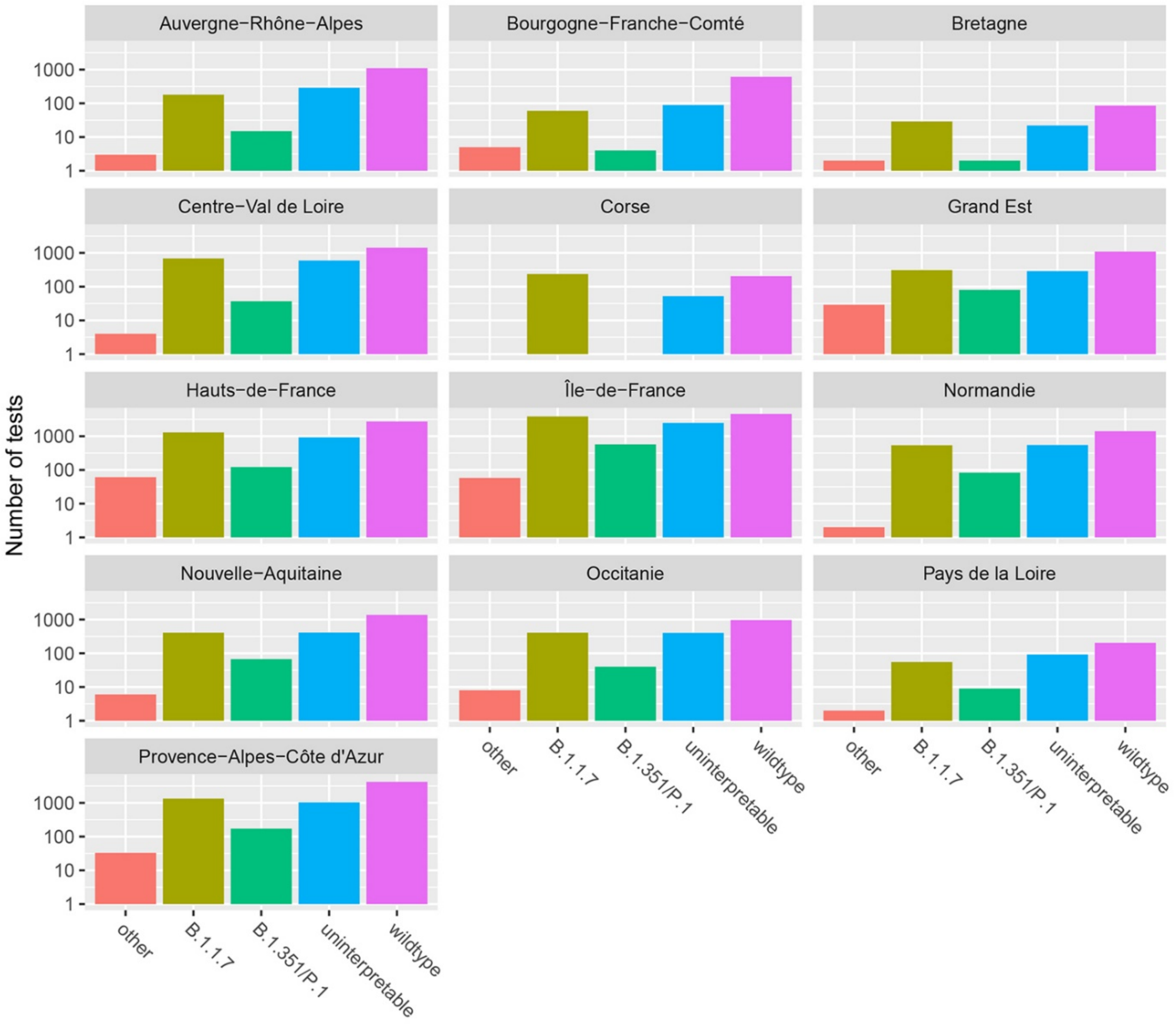

Appendix 2 Figure 1. Results of severe acute respiratory syndrome coronavirus 2 variant-specific reverse transcription PCR test results per geographic region, France, January 26–February 16, 2021.

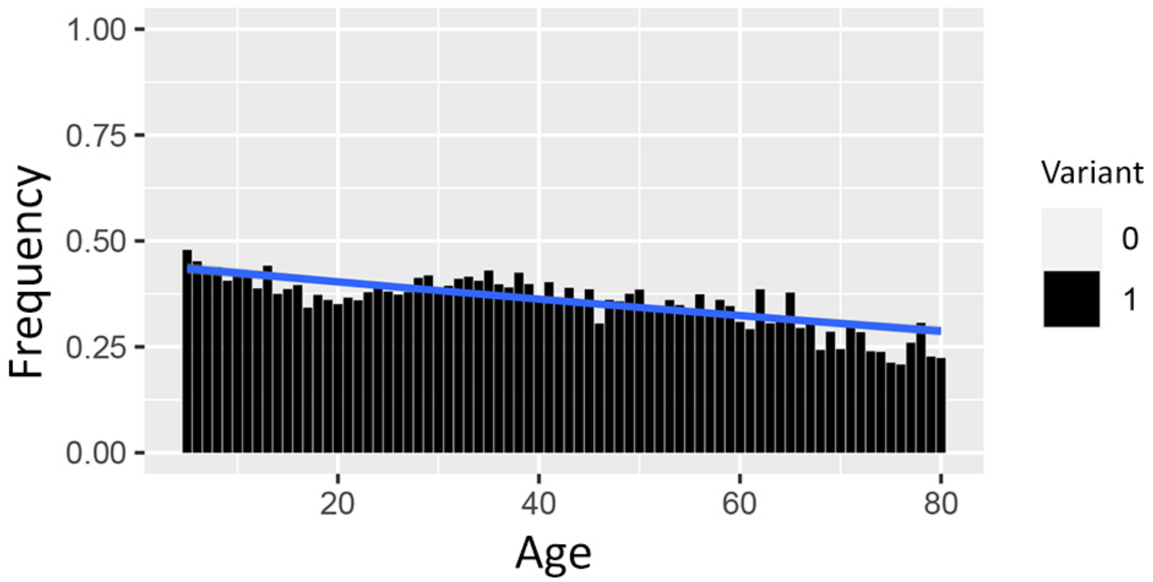

Appendix 2 Figure 2. Proportion of severe acute respiratory syndrome coronavirus 2 infections caused by variants as a function of age, France, January 26–February 16, 2021. The blue line is the trend obtained using a univariate generalized linear model. Uninterpretable tests as well as age classes <5 years or >80 years are not shown.

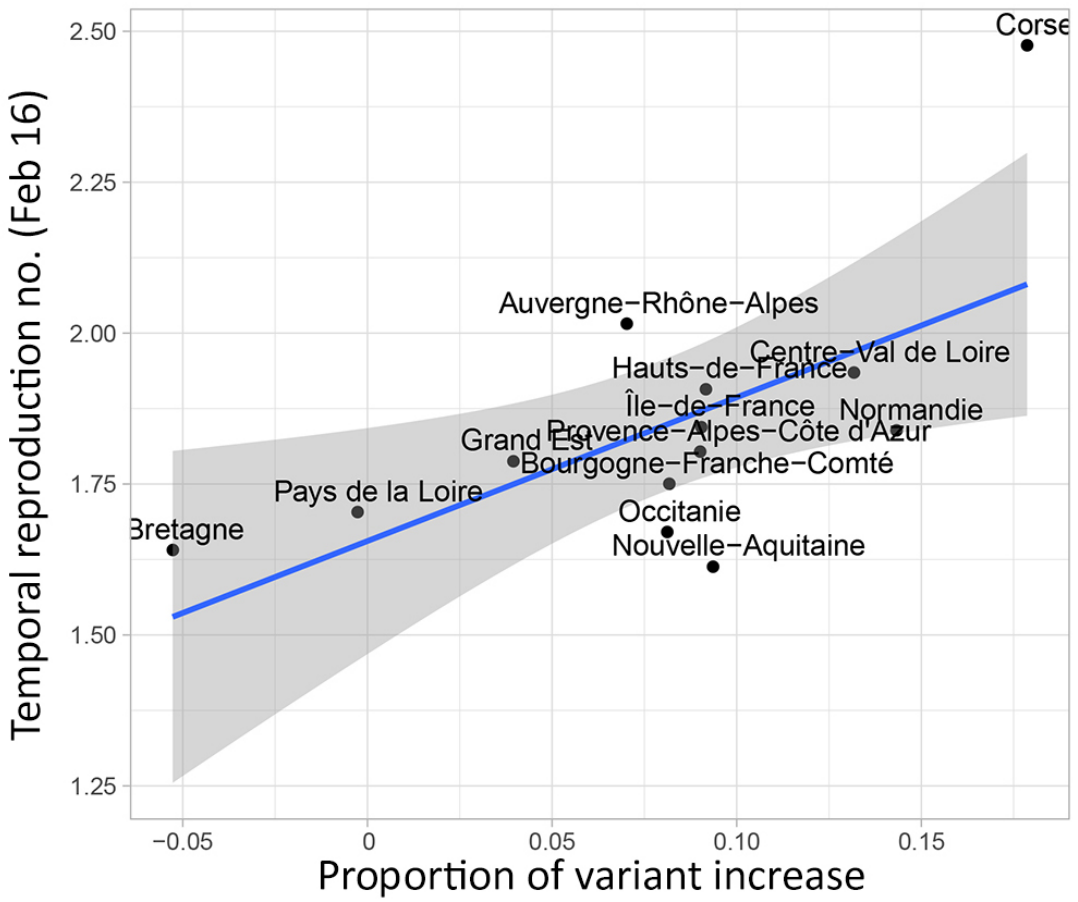

Appendix 2 Figure 3. Regional reproduction number (R_t) as a function of the estimated increase in severe acute respiratory syndrome coronavirus 2 variant frequency, France, January 26–February 16, 2021. The dashed line shows the output of a univariate linear model.

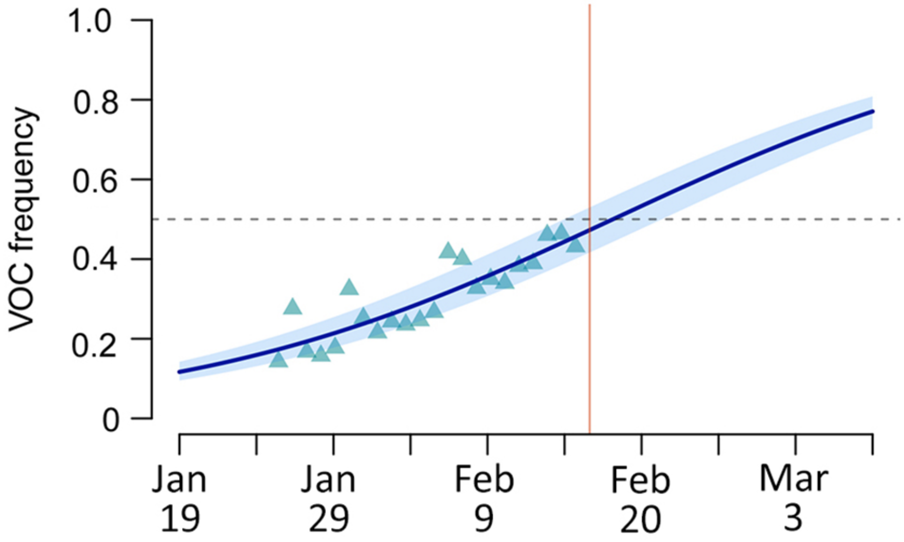

Appendix 2 Figure 4. Severe acute respiratory syndrome coronavirus 2 variants frequency, assuming that uninterpretable tests are all caused by wild-type strains, France, January 26–February 16, 2021. The value indicates the transmission spread advantage of the variants and its 95% CI.

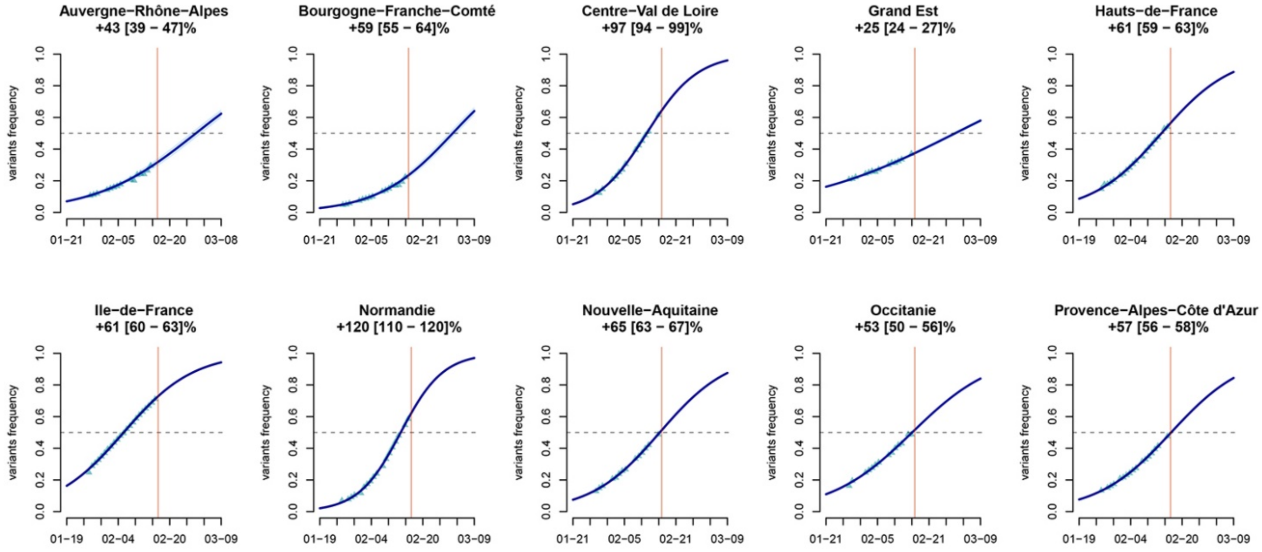

Appendix 2 Figure 5. Logistic growth at the regional level in study of rapid spread of severe acute respiratory syndrome coronavirus 2 variants, France, January 26–February 16, 2021. The dots indicate the generalized linear model–fitted values, and the line is the output of the logistic growth model estimation. The vertical orange bar indicates the date of the analysis. The caption on the top of the figures indicate the estimated transmission advantage of the variants (with respect to the wild-type reproduction number) and its 95% CI.
